# Some Modern Methods of Local Treatment of the Stomach and the Intestines*Read at the twenty-second annual meeting of the Western Texas Medical Association.

**Published:** 1899-05

**Authors:** H. Leonards

**Affiliations:** New Braunfels, Texas


For Texas Medical Journal.

### Some Modern Methods of Local Treatment of the Stomach and the Intestines.*

BY H. LEONARDS, M. D., NEW BRAUNFELS, TEXAS.

The treatment of the diseases of the intestinal tract entered a new era with the introduction of the stomach tube as a therapeutic agent. Before this date, the physician, not knowing much of the pathological contents of the diseased organ, was satisfied to prescribe a stomachic mixture, or some other preparation, like pepsin, pancreatin or muriatic acid, which are, so physiology had taught him, the necessary normal adjuvants in the stomachical or intestinal digestion. By exploring the diseased stomach and evacuating its contents and examining the same, we learned the futility of the treatment formerly in vogue.

At this period the use of antiseptics had been followed by phenomenal success in the various diseases caused by the presence of septic or purulent material. Discovering in the contents of the diseased stomach or intestines septic and fermented material, the

*Read at the twenty-second annual meeting of the Western Texas Medical Association.

antiseptic treatment had of course to be tried in these conditions. The various antiseptics and antiferments came in vogue in the treatment of the diseases of the stomach and intestines, and were tried judiciously as to their real value, but they were found to be, to say the least, inadequate.

Discouraged with the results of this line of treatment, we took refuge in mechanical methods. The first step in this direction was made, as I have said above, with the discovery of the stomach tube. The periodical emptying of a dilated and atonic stomach by the aid of the tube, as the first mechanical therapeutic measure, is surely entitled to be called a great advance in our therapeutics.

There has been of late a lively interest in the mechanical treatment of the intestinal tract. By enthusiastic workers in this specialty other contrivances have been devised for varied purposes. Of these methods, I may enumerate four, which are of recent date, and not yet accepted by the general practitioner. These are:

(1) The gyromele, or the stomach brush.
(2) The needle douche.
(3) The stomach and intestinal nebulizer.
(4) The hot water rubber bag.

My first practical experience with these new methods I got in the clinic of Prof. Turk, of Chicago. I take liberty in explaining to you, in a few words, their value, and in demonstrating their mode of application.

The gyromele, or the revolving brush, consists of a sponge attached to the end of a cable at the end of which is an apparatus for the purpose of revolving the sponge. The revolving apparatus works somewhat like an ordinary egg-beater. The sponge is passed into the stomach in the same way as the stomach tube, after some soap and water has been introduced. Then rapid revolutions are produced for some minutes. By these means food remnants and mucus of glue-like consistency are readily detached from the walls of the stomach, and can be removed by the tube. It is evident that this is of great value, as the removal of this mucous coat deprives the micro organisms of the nutrient media in which they develop. It furthermore serves as an excellent method of internal massage and vascular stimulant, and can even be used with advantage in combination with the electric current. The use of the gyromele recommends itself in the same way in diseases of the colon. It is not accompanied with any danger except in cases of ulcers or carcinoma ventriculi, where one has to be cautious on account of possible hemorrhage.

The needle douche consists of a double tube connected with a needle spray extending, when introduced, to the cardiac end of the stomach. It gives a fine spray to the entire wall. The necessary pressure in the douche is produced by compressing with an ordinary bulb the air in a glass partly filled with water. The fluid does not distend the stomach as in lavage or syphon, as it immediately returns through the larger tube. The indications for this apparatus and its effects explain themselves, and need no further consideration.

The nebulizer consists of a double tube in connection with an atomizer. With this apparatus the various antiseptics, like formalin, oil of cloves, oil of cinnamon, and astringents, can be brought in form of a fine cloud into the stomach or the intestines.

The stomach gets distended by the air introduced through the tube, and the whole wall is covered with a fine coating of the antiseptic. The contraction of the stomach presses the air out through the other side of the double tube. This rythmic distension and contraction serves as a massage, or as a kind of pneumatic gymnastic, giving strength and tone to the weakened muscles of the stomach. The nebulizer can also be used in diseases of the colon, especially in dilatation with lack of peristalsis. Experiments have shown that by these means the entire colon can be inflated, and thus the mucous membranes be coated with a thin layer of an antiseptic or astringent.

The rubber bag apparatus consists of a double tube, at one end connected with a rubber bag, at the other end with a hot water irrigator. The water is used at a temperature of 125° to 130°. With the aid of the rubber bag we are able to use water at this high temperature, which we could not do otherwise. The rubber protects the mucous membrane of the stomach against direct contact and scorching; 110° would be the highest allowable temperature in bringing the hot water directly into the stomach.

The collapsed bag is introduced into the stomach like the ordinary tube. The hot water passes through one tube into the bag, and after filling the same to the fullest extent, runs out through the other tube. The hot water, in passing this way through the bag inside of the stomach, loses a great amount of heat. The reduction of the temperature of the water is greater and goes on more rapidly than if the bag would be put in cold water, a fact which is difficult to explain by physiological laws.

It is evident that the continuous action of such high temperature right inside of the stomach must result in marked vasomotor stimulation. The time of the treatment is about twenty minutes. The direct effect is a very prompt one. The whole surface of the body gets into a glow, and the hands and feet become warm. This indicates that the visceral congestion is reduced and the circulation of the blood in the whole body equalized. It is easy to understand what excellent therapeutic effect this measure must have in anemic and debilitated patients with chronic gastritis, dyspepsia, dilatation of the stomach, etc., which cases are always accompanied with more or less passive congestion in the internal organs.

These are the few new and modern methods in the treatment of the intestinal tract which I have the pleasure of bringing before this Association. I have seen these methods used extensively for a number of weeks in Prof. Turk's clinic in Chicago. Dozens of patients were treated daily by these methods, and the direct results seemed to me, so far as I could judge, to be very satisfactory. I remember especially three anemic and sickly looking women suffering from dilatation of the stomach, with all the characteristic symptoms. They were treated at the same time with the gyromele, followed with the hot water bag. Once every week the blood was examined, which showed a gradual but steady increase in the number of red blood corpuscles with every examination. In the sixth week the increase amounted to about a million on the average in the cubic millimeter. At the same time the general appearance of the patients improved, and the former symptoms disappeared. Considering that these cases got no other treatment, besides the one named above, the result, I must admit, was very flattering.

My experience in private practice in the use of these methods, I am sorry to say, is a rather limited one. Cases of gastric and intestinal trouble, severe enough to justify such local treatment, are comparatively rare in a country practice, and if they come under the physician's treatment, it is seldom possible to make them undergo such a treatment, which has to be regular, and continued for at least a number of weeks. Quite different is this in a city practice. Here, I believe, these methods will be employed to the great satisfaction of the physician, as well as the benefit of his patient.

